# NAC and Vitamin D Improve CNS and Plasma Oxidative Stress in Neonatal HIE and Are Associated with Favorable Long-Term Outcomes

**DOI:** 10.3390/antiox10091344

**Published:** 2021-08-25

**Authors:** Dorothea D Jenkins, Hunter G Moss, Truman R Brown, Milad Yazdani, Sudhin Thayyil, Paolo Montaldo, Maximo Vento, Julia Kuligowski, Carol Wagner, Bruce W Hollis, Donald B Wiest

**Affiliations:** 1 Division of Neonatology, Department of Pediatrics, Medical University of South Carolina, 10 McClennan Banks Drive, Charleston, SC 29425, USA; wagnercl@musc.edu (C.W.); hollisb@musc.edu (B.W.H.); 2 Center for Biomedical Imaging, Department of Radiology, Medical University of South Carolina, Charleston, SC 29425, USA; mossh@musc.edu (H.G.M.); brotrr@musc.edu (T.R.B.); yazdani@musc.edu (M.Y.); 3 Centre for Perinatal Neuroscience, Imperial College London, London W12 0HS, UK; s.thayyil@imperial.ac.uk (S.T.); p.montaldo@imperial.ac.uk (P.M.); 4 Neonatal Research Group, Health Research Institute Hospital La Fe, 46026 Valencia, Spain; maximo.vento@uv.es (M.V.); julia.kuligowski@uv.es (J.K.); 5 Department of Clinical Pharmacy and Outcomes Sciences, College of Pharmacy, Medical University of South Carolina, Charleston, SC 29425, USA; wiestdb@musc.edu

**Keywords:** N-acetylcysteine, vitamin D, neonatal HIE, oxidative stress, MRS

## Abstract

N-acetylcysteine (NAC) and vitamin D provide effective neuroprotection in animal models of severe or inflammation-sensitized hypoxic ischemic encephalopathy (HIE). To translate these FDA-approved drugs to HIE neonates, we conducted an early phase, open-label trial of 10 days of NAC (25, 40 mg/kg q12h) + 1,25(OH)_2_D (calcitriol 0.05 mg/kg q12h, 0.03 mg/kg q24h), (NVD), for pharmacokinetic (PK) estimates during therapeutic hypothermia and normothermia. We paired PK samples with pharmacodynamic (PD) targets of plasma isoprostanoids, CNS glutathione (GSH) and total creatine (tCr) by serial MRS in basal ganglia (BG) before and after NVD infusion at five days. Infants had moderate (*n* = 14) or severe HIE (*n* = 16), funisitis (32%), and vitamin D deficiency (75%). NVD resulted in rapid, dose-responsive increases in CNS GSH and tCr that correlated positively with plasma [NAC], inversely with plasma isofurans, and was greater in infants with lower baseline [GSH] and [tCr], suggesting increases in these PD markers were titrated by neural demand. Hypothermia and normothermia altered NAC PK estimates. NVD was well tolerated. Excluding genetic syndromes (2), prolonged ECMO (2), lost-to-follow-up (1) and SIDS death (1), 24 NVD treated HIE infants have no evidence of cerebral palsy, autism or cognitive delay at 24–48 months. These data confirm that low, safe doses of NVD in HIE neonates decreased oxidative stress in plasma and CNS, improved CNS energetics, and are associated with favorable developmental outcomes at two to four years.

## 1. Introduction

Therapeutic hypothermia provides significant neuroprotection for half of neonates with uncomplicated hypoxic ischemic encephalopathy (HIE), but not in more severe HIE or HIE complicated by chorioamnionitis [1,2,3,4,5,6]. Oxidative stress is both an early and persistent contributor to pathology in HIE [7,8,9,10,11]. Glutathione (GSH) is the major intracellular antioxidant which scavenges reactive oxygen species (ROS) and is essential for cell survival [12,13]. Evidence in animal models and humans indicate that oxidative stress depletes reduced GSH within two hours, which is not mitigated by hypothermia alone [14,15,16]. Increased production of superoxide and lipid peroxides persists for days to weeks after moderate to severe HI/stroke, with significant depletion of GSH in the striatum, hippocampus, cortex, and cerebellum [17,18,19]. Additionally, the decrease in GSH potentiates glutamate toxicity and FAS-activated cell death [20,21,22,23]. Increasing intracellular antioxidant reserves in the brain very early after HI may be key to halting progression of neural cell death and improving neuroprotection for HIE infants who do not respond to hypothermia. 

Oxidative stress is a trigger for many inflammatory cascades, and early restoration of normal intracellular redox potential moderates these same pathways. Increasing the CNS pool of available GSH in the metabolically active basal ganglia (BG) may improve neuroprotection after significant neonatal HIE [24]. N-acetylcysteine (NAC) effectively mitigates oxidative stress in animals and humans [9,25,26]. NAC crosses the blood–brain barrier, provides the rate-limiting substrate for GSH synthesis, increases intracellular GSH concentrations and improves cell survival in animal models and humans with multiple etiologies of oxidative stress [9,27,28,29,30,31,32,33,34,35,36]. In preclinical work, we previously demonstrated that NAC plus hypothermia improved outcomes over hypothermia alone in neonatal female, but not male, rats subjected to severe HI [37]. When 1,25(OH)_2_D (calcitriol) was co-administered with NAC and hypothermia, male neonatal rats also showed decreased neuroinflammation and improved neuroprotection [16]. Active vitamin D is a neuro-steroid involved in myelination, neuroplasticity and normal development [38,39,40]. While anti-inflammatory effects are well described, 1,25(OH)_2_D also induces synthesis of glutathione reductase, the enzyme responsible for regeneration of GSH from oxidized glutathione disulfide, and thereby increases GSH [41,42,43,44,45]. We postulated that NAC plus 1,25(OH)_2_D (NVD) would synergistically increase GSH, decrease oxidative stress and improve developmental outcomes in human neonates with severe and complicated HIE [16,46,47]. 

Therapeutic development of antioxidants in clinical translational neuroscience has been hampered by a lack of careful measurement of biomarkers in target tissue to ensure dosing for pharmacodynamic effect (PD). Biomarkers of oxidative stress include reduced glutathione (GSH) and protein and lipid peroxidation products, which are usually measured in blood. MR spectroscopy (MRS) can directly measure CNS GSH in vivo after injury or treatment [14,29,48,49,50,51]. Therefore, we designed this translational study to determine (1) the doses of NAC and 1,25(OH)_2_D that effectively mitigate oxidative stress in CNS and blood in HIE neonates treated with hypothermia, (2) the duration of NAC and 1,25(OH)_2_D effect on CNS metabolites and any dose-limiting side effects during hypothermia or normothermia, and (3) the association of improved oxidative stress biomarkers with neurodevelopmental outcomes at >2 years of age.

In a previous rapid communication, we reported our validation of MRS quantification of GSH in this cohort of convalescing HIE infants, with mean BG [GSH] of 1.6 ± 0.2 mM on day of life (DOL) 5 after hypothermia treatment, which is markedly lower than 2.5 ± 0.8 mM previously reported in healthy term neonates [52]. We also reported that NAC with or without calcitriol rapidly and significantly increased [GSH] in the BG to 1.93 ± 0.23 mM (*p* < 0.0001) on DOL 5–6 [15]. 

In this manuscript, we report the results of the complete clinical trial of NAC and calcitriol (NVD) in neonates with HIE undergoing hypothermia. We quantified plasma lipid peroxidation products and CNS metabolomics with paired plasma NVD concentrations before and after NVD and employ novel, serial MRS to show PD dose response and duration of GSH response in the BG, a major CNS target for neuroprotection in HIE. We show that NAC and calcitriol were safe and determine the dose tolerability and pharmacokinetics (PK) during hypothermia and normothermia. Finally, we report on developmental outcomes of study participants from two to four years of age. 

## 2. Materials and Methods

### 2.1. Study Design Overview 

We enrolled 30 neonates with moderate to severe HIE receiving hypothermia in this Institutional Review Board (IRB)-approved open-label, early phase study (NCT 04643821). We administered intravenous (iv) NAC and calcitriol (NVD) daily for 10 days. We obtained blood samples for NVD PK estimates and oxidative stress markers around the first dose and during 72 h of hypothermia (HT) and around the 10th–11th dose on DOL 5–6 during subsequent normothermia (NT). We paired plasma PK and oxidative stress samples around the 10th or 11th doses with MRS before and after NVD infusion on DOL 5–6, to determine CNS PD dose response in [GSH] and duration of the CNS effect. We were powered to detect 20% [GSH] change of NVD with 20 paired MRS datasets. Our primary safety outcomes were hypotension (NAC) and hypercalcemia (calcitriol). MRS quantification was blinded to dose and condition.

#### 2.1.1. Consent and Enrollment

This study was approved by the Medical University of South Carolina Institutional Review Board. We approached parents within 6 h of birth and obtained written informed consent according to the Declaration of Helsinki, prior to enrolling 30 consecutive neonates with HIE in MUSC’s Neonatal Intensive Care Unit from 2014 to 2017. All infants had moderate to severe HIE by modified Sarnat staging [53,54], and qualified for cooling for 72 h according to standard HIE criteria: Gestational age ≥ 34 weeks, and they had one factor indicating an acute sentinel event (cord or baby pH ≤ 7.0, base deficit ≥ −13, Apgar score ≤ 5 at 10 min, continued resuscitation after 5 min due to absence of respiratory effort), and two signs of stage 2/3 encephalopathy [55]. 

#### 2.1.2. Standard of Care: HIE Protocol and Hypothermia Treatment

Infants were cooled to 33–33.5 °C T_r_ for 72 h (Criticool™, Belmont Medical Technologies, Billerica, MA), and rewarmed at 0.2 °C/h. Infants received nothing by mouth through rewarming other than oral care with breast milk. Parenteral nutrition without cysteine, but with vitamin D_3_ 400 IU/day and lipids (0.5–2 gm/kg/d), was administered during hypothermia. Feeds were introduced if clinically stable on DOL 4–5. Infants received IV morphine 0.02 mg/kg every 4 h for comfort during hypothermia that was increased as clinically required in infants with pulmonary hypertension (PPHN). Standard monitoring during hypothermia included serum lactate, ionized and total calcium, electrolytes, clotting studies, liver function tests, cardiac and renal panels, and circulating cell counts. Continuous 20 montage video EEG was monitored from admission until completion of rewarming. Seizures were defined as electrographic seizure activity. Head ultrasound with Doppler blood flow of three cerebral arteries was obtained within 12 h of admission using a Philips Epic 5 scanner (Koninklijke Philips N.V., Amsterdam, The Netherlands) with a 9–4 MHz curvilinear transducer, and we recorded the average resistive index and time average maximum velocity. Echocardiograms were obtained in all infants. PPHN was defined as evidence of right to left intra- or extracardiac shunting through the patent ductus arteriosus. An MRI and MRS were obtained on day of life 5–6 after rewarming, on a 3T Siemens Skyra, using standard sequences for T1, T2, ADC, and DKI. For MRS, single voxels were placed in the left BG and right frontal white matter. MRI scans were read by a single neuroradiologist (MY).

#### 2.1.3. NAC and Calcitriol Infusion

The study drugs were administered by IV within 4–9 h after birth and continued until DOL 10 or discharge. NAC was infused over 60 min, and calcitriol by IV push. For the first 20 participants the NAC dose was held constant at 25 mg/kg/dose every 12 h while determining the optimal dose and timing of calcitriol. For the next 10 participants, the NAC dose was increased to 40 mg/kg, keeping the calcitriol dose constant. Treatment groups were NAC 25 mg/kg/dose + calcitriol 0.05 mcg/kg/dose iv q12h (*n* = 10), NAC 25 mg/kg/dose q12h + calcitriol 0.03 mcg/kg/dose q24h (*n* = 10), or NAC 40 mg/kg/dose q12h + calcitriol 0.03 mcg/kg/dose q24h (*n* = 10). Both study drugs were held if an infant had untreated/refractory hypotension at time of dosing (mean BP < 40 mmHg). Calcitriol was held for ionized calcium (iCa^++^) > 1.3 mmol/L, and we limited calcium in parenteral nutrition. An early calcitriol PK analysis estimated serum calcitriol half-life to be 28.2 h during hypothermia and mean peak [1,25(OH)_2_D] = 263 ± 72 pmol/L (first dose), increasing to 434 ± 146 pmol/L (sixth dose), indicating continued accumulation with 0.05 mcg/kg/dose q12h. Subsequently the calcitriol dose was decreased to 0.03 mcg/kg/dose q24h (*n* = 20), but still within the range of effective doses in our preclinical studies [16]. 

### 2.2. Magnetic Resonance Spectroscopy

#### 2.2.1. MRS Protocol

**Figure 1:**
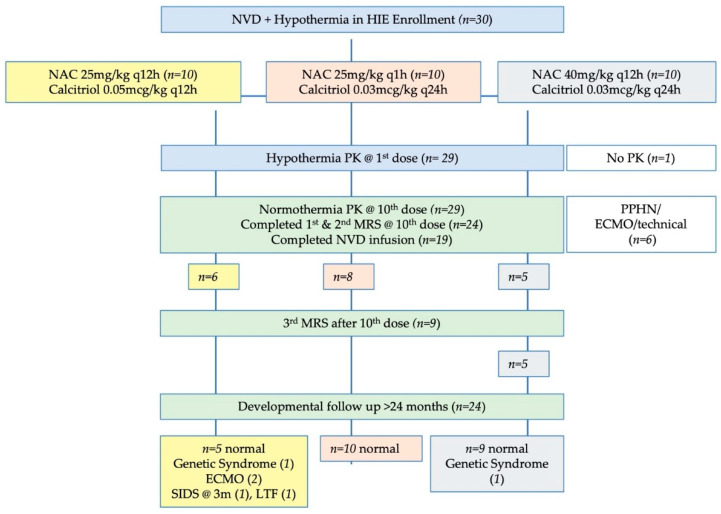
Study flow diagram. Note that the number of infants with developmental follow-up flows from the original number of infants in each dosing group, not just the infants with complete NVD infusions or complete MRS data. The number of infants with useable data for each MRS metabolite may vary slightly from these numbers due to MRS quality controls for spectral fit which are unique to each metabolite.

On DOL 5–6 after rewarming, clinically stable HIE neonates had MRS scans with paired blood samples for NVD and lipid peroxidation products before and after the 10th or 11th NVD dose, using a single voxel placed in either the BG or frontal white matter, with stimulated echo acquisition mode (STEAM), echo time (TE) 20 ms, and point resolved spectroscopy (PRESS) TE 270 ms on a Siemens 3T Skyra, as previously described [15]. Infants received both NAC and calcitriol, or only NAC if the calcitriol dose was held or not given at that time with 24 h dosing, as scans had to be conducted at night. The neuroimaging protocol (Figure 1) consisted of: (1) first MRS immediately preceding NVD dose administration (trough); (2) calcitriol and NAC infusion during the clinical MRI; (3) second MRS immediately after clinical MRI (12–30 min after NVD infusion, peak); (4) third delayed MRS (2–6 h after infusion as the clinical schedule allowed, post-peak) to estimate the duration of NAC effect on [GSH] in the CNS; (5) MRS/diffusion imaging obtained between 10–40 d (convalescent). Sedation with low dose morphine or lorazepam was administered for clinical indications during acute scans. Scans required 90 min for the acute protocol, 12 min for the delayed scan. The PI (neonatologist) and neonatal intensive care unit nurse were present during all scans.

#### 2.2.2. LCModel Data Fitting

MRS spectra were fit with LCModel by an investigator blinded to subject and dose, using a custom basis set that included GSH with a cysteinyl peak at 2.95 ppm, as well as standard metabolites: total Choline (tCho: GPC+PCho), total creatine (tCr: Cr+PCr), total N-acetylaspartate (NAA: NAA+NAAG), myoinositol (mIns), glutamate and glutamine (GLX)] [15]. Absolute metabolite concentrations were determined with water normalization from the unsuppressed water scan. Representative 3T MRS of the GSH peak at 2.95 ppm and validation of quantification has been previously published [15]. We excluded spectra for low signal/noise < 5, or coefficient of variation > 15% by LCModel fit. 

### 2.3. Pharmacokinetic Studies

#### 2.3.1. Drug Concentrations

Blood samples were collected for HT and NT PK: before and 0.5 h, 1 h, 11.5 h after first (HT) and tenth (NT) NAC/NVD doses, and for steady-state trough concentration (Cmin_ss_) prior to sixth (HT) and thirteenth doses (NT). Blood was collected in sodium EDTA (NAC) and plain tubes (vitamin D), and plasma/serum was aliquoted and frozen at −80 °C until assay. CSF obtained at 24 h with the peak NVD blood sample was frozen at −80 °C until assay. Total plasma NAC concentrations were determined using a modified, sensitive and specific HPLC method with a limit of detection of 0.15 mmol/L [56]. Serum 25(OH)D & 1,25(OH)_2_D concentrations were measured for 23 infants by RIA with detection limits of 2.5 mol/L for 25(OH)D and 36 pmol/L for 1,25(OH)_2_D [57]. Frozen aliquots of serum for 20 infants were shipped on dry ice for analysis of isoprostanoids markers of oxidative stress by LC-MS, as previously reported [18]. 

#### 2.3.2. PK Analyses

Non-compartmental modeling methods (PK Solutions 2.0; Summit Research Services, Montrose, Colorado) generated PK parameters for NAC and 1,25(OH)_2_D: terminal elimination half-life (t_1/2_), volume of distribution (Vd), total body clearance (CL) and area under the concentration curve (AUC). Statistical comparisons between doses or HT and NT were conducted using a paired or unpaired 2-sided Student *t* test, with significant *p*-value < 0.05. (Graphpad Software 5.00, San Diego, CA, USA). 

#### 2.3.3. Developmental Follow-Up Testing

We performed standard developmental testing in high-risk follow-up or developmental clinics for most participants between 18–36 months of age. This battery of tests includes the Peabody Developmental Motor Scales, which have a high correlation with the Bayley III motor scores (*r* = 0.79–0.88 for fine, and gross/total motor scores, respectively [58]; Cognitive adaptive test (CAT) and Clinical linguistics and auditory milestone scale (CLAMS), which are both sensitive and specific for cognitive delays defined as the Bayley cognitive scaled score < 70 [59] and the Modified Checklist for Autism in Toddlers (MCHAT). Parental reports of development by phone were also obtained at 4 years of age to discern any intervening issues with language or speech. Two families moved out of the area and only provided parental reports of development and the use of special services.

#### 2.3.4. Statistical Analysis

For 24 subjects with CNS metabolite concentrations regardless of completion of NVD infusion (intent-to-treat), two-tailed paired t-tests compared changes in metabolite concentrations in HIE neonates pre- and post-infusion with significance *p <* 0.05 (IBM SPSS^®^ v.25, Armonk, NY, USA). ANOVAs were used to compare repeated measures of non-linear MRS metabolite concentrations for three time points within a subset of 10 subjects, with pairwise comparisons and Bonferroni correction. Partial eta squared values are reported for attributing the variance in metabolite concentration to time of scan around NVD infusion. Independent variables of NAC dose and sex were included in analyses. We performed both intent-to-treat analyses of CNS metabolites and efficacy analysis (infants who received full dosing) to account for infusion problems and lack of reliable dose delivery during some MR scans. Pearson’s or Spearman’s correlation coefficients were generated for comparisons of plasma NAC and lipid peroxidation products, and CNS metabolite concentrations, as appropriate. 

## 3. Results

### 3.1. Clinical Characteristics

We enrolled 30 neonates with moderate to severe HIE receiving hypothermia in this IRB-approved open-label, early phase study (Figure 1). Study drugs were administered within 4–9 h after birth and continued through 10 days of life through a central or peripheral IV line. 

Clinical demographic data of the neonatal HIE cohort are presented in Table 1. There were twice as many males as females. Infants exhibited neurologic signs of moderate to severe encephalopathy prior to enrollment and were hypothermic at first study drug administration. Out of the 19 placentas sent to pathology we demonstrated acute chorioamnionitis with funisitis in one third of HIE infants. One placenta showed fetal vasculopathy with thrombosis and many avascular villi. The numbers of infants in the worst quartiles for pH (6.5–6.8) and base deficit (>−20) are also given.

Six infants had PPHN prior to enrollment and four were treated with inhaled nitric oxide. Two of these infants required prolonged and complicated courses of extracorporeal membrane oxygenation (ECMO): one was placed on veno-venous by-pass and deteriorated over the next 24 h requiring conversion to veno-arterial bypass with overwhelming sepsis and pneumonia. Study drugs were held for 41 h during these events. The second infant was placed on ECMO within a few hours of birth and did not receive the first dose of study drug until blood pressure was stable on bypass. This infant later suffered an emergent circuit change due to circuit thrombosis with subsequent major pulmonary and CNS hemorrhages with hypoxia (1). Two genetic disorders were diagnosed after treatment completion (Prader Willi and congenital myasthenia gravis). 

Resistive indices on Doppler ultrasound (*n* = 28) within 24 h of HIE birth were mean RI = 0.72 ± 0.11(0.53–0.94) in the anterior cerebral artery, 0.73 ± 0.11 (0.53–0.90) in the middle cerebral artery, and 0.74 ± 0.10 (0.54–1.0) in the basilar artery. Abnormal RI (<0.65 or >0.8) were noted in 13 infants in the middle cerebral and basilar arteries and 15 infants in the anterior cerebral artery [60,61].

MRI brain parenchymal signal abnormalities were present in 10 infants, largely demonstrating small foci of periventricular signal abnormality characterized by subcentimeter foci of T1 and T2 shortening with associated restricted diffusion (Table 1). Other imaging abnormalities included one patient with right sided subcentimeter cortical hemorrhages, one patient with left subinsular white matter injury, one patient with subcentimeter focus of T2 hyperintensity in the right basal ganglia, one patient with a subcentimeter right cerebellar hemorrhage, and one patient with left middle cerebral artery and additional multifocal small bilateral cortical infarctions. Among the 10 infants with minor abnormal parenchymal findings, four had small volume intraventricular hemorrhage. Among the patients with no brain parenchymal signal abnormalities, one patient had trace intraventricular hemorrhage. Seventeen infants had no identifiable abnormalities on their conventional MRIs. 

### 3.2. NVD Safety and Tolerability

NAC 25 and 40 mg/kg/dose q12h were well tolerated without serious adverse events. We held NAC and calcitriol doses for urgent cannulation for ECMO per IRB-approved protocol. Patients on ECMO received an NAC loading dose to account for increased Vd of the ECMO circuit. Calcitriol dose was not altered.

We held 26 calcitriol doses for elevated iCa^++^ (mean iCa^++^ = 1.42 ± 0.06 mM), which were not related to dose or interval. iCa^++^ was elevated in five out of ten infants receiving calcitriol 0.05 mcg/kg q12h (1.46 ± 0.02 mM), and 18 out of 20 infants receiving calcitriol 0.03 mcg/kg q24h (iCa^++^ 1.41 ± 0.07 mM). Many of the infants had calcitriol doses held while on full feeds, when ionized calcium concentrations generally increased. Therefore, the mean duration of calcitriol dosing was 5.7 ± 2.1 days. We observed no clinical effects of mildly elevated ionized calcium, which was managed by withholding calcitriol dosing and/or decreasing calcium in parenteral nutrition. Elevated iCa^++^ were not associated with elevated total serum calcium concentrations (all < 12 mg/dL).

### 3.3. NAC and Vitamin D Pharmacokinetics 

#### 3.3.1. NAC PK

**Figure 2:**
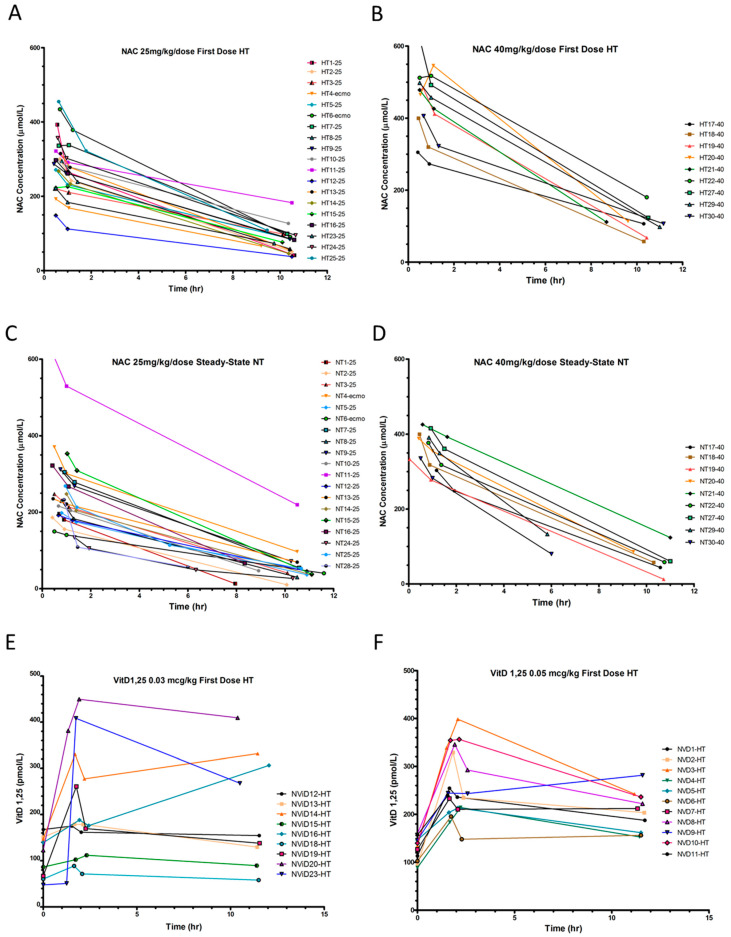
Plasma NAC and serum 1,25(OH)_2_D concentrations and pharmacokinetics. (**A**,**B**) Plasma NAC concentrations during hypothermia (HT) and (**C**,**D**) normothermia (NT), with NAC (**A**,**C**) 25 mg/kg/dose and (**B**,**D**) 40 mg/kg/dose. (**E**,**F**) 1,25(OH)_2_D serum concentrations during HT with calcitriol (**E**) 0.03 mcg/kg/dose, (**F**) 0.05 mcg/kg/dose.

We found significant differences in NAC PK parameters between HT and NT periods, with lower clearance and longer half-life during hypothermia (Table 2, Supplementary Table S1, Figure 2A–D). Steady-state NAC trough (Cminss) concentrations were low during both periods with q12h dosing. NAC PK did not significantly differ between calcitriol dosing groups or when ECMO patients were excluded from analysis. 

#### 3.3.2. 25(OH)D and 1,25(OH)_2_D Serum Concentrations at Birth and Pharmacokinetics

At birth, 78% of 23 HIE neonates sampled were vitamin D insufficient [25(OH)D < 75 nmol/L], 61% were vitamin D deficient [<50 nmol/L], and 30% were severely deficient [<25 nmol/L], similar to our previous HIE cohort [57]. There were significant differences in 25(OH)D serum concentrations at birth by race but not by sex. Black and Hispanic infants had mean 25(OH)D 25 ± 10 nmol/L vs. white infants 62.5 ± 25 nmol/L (*p* = 0.00025). Furthermore, all black/Hispanic infants were 25(OH)D deficient at birth, range (12.5–45 nmol/L). Among white infants, 31% were deficient, 31% were insufficient, and 38% were 25(OH)D sufficient. 25(OH)D serum concentrations did not increase with 400IU vitamin D_3_ added to daily parenteral nutrition or enteral feeds.

Mean 1,25(OH)_2_D serum concentrations were 48 ± 16 pg/mL shortly after birth and did not vary by race or sex. Serum [1,25(OH)_2_D] increased with the first calcitriol dose by 80.6 ± 25.2 pmol/L (0.03 mcg/kg) and 143.5 ± 62.2 pmol/L (0.05 mcg/kg), but show considerable variability in PK, particularly with the 0.03 mcg/dose (Figure 2E,F). Many patients had concentrations at 60 min that were higher than “peak” concentrations at 30 min, precluding PK estimate calculations. This may have been due to the small dose volume being administered, continued release from vitamin D binding protein [62], or a prolonged equilibration phase. Calcitriol was frequently held during normothermia for elevated iCa^++^. Therefore, we report calcitriol PK for only six patients receiving 0.05 mcg/kg during HT (Figure 2F, Table S2), which prevented any meaningful comparisons between doses, or HT and NT periods.

#### 3.3.3. NAC and Vitamin D concentrations in CSF

We measured NAC concentration in the CSF in five infants (mean 17.2 ± 23.9 mmol/L, range: 6.1–61.3 mmol/L) within 7–25 min after the second NAC dose (Supplementary Table S1). Compared with the plasma obtained at the same time as CSF, secretion of NAC into CSF was between 1–21% of peak plasma [NAC]. 1,25(OH)_2_D was not detectable in any CSF sample.

### 3.4. MRS Metabolite Changes with NVD Infusion

NVD was infused during the routine MRI on DOL 5–6, and MRS scans were performed at 0 h before infusion, at 0.5 h, and 2–6 h after infusion to study the PD time course of [GSH] change in CNS. We obtained adequate MRS spectra and metabolite data in 23–24 neonates at two time points depending on metabolites, and in 10 patients at three time points. For efficacy analyses we excluded MRS if there were problems with the IV or infusion pump resulting in incomplete infusion of NVD prior to second scan (*n* = 5), and significant delay outside of dosing windows by emergent clinical cases (*n* = 1). As previously reported, three infants had no NAC infused prior to the second scan and had no increase in [GSH] [15]. One infant had left parietal perinatal stroke, and a voxel was placed in the diffusion-restricted stroke area in addition to the BG.

#### 3.4.1. Dose Responsive Increase in [GSH] with NVD Infusion in Basal Ganglia

**Figure 3:**
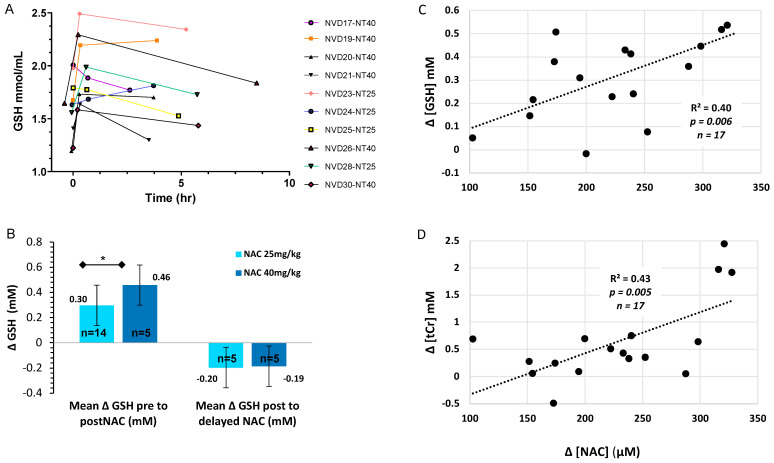
CNS GSH changes over time and by NAC dose. (**A**) [GSH] in BG of the 10 HIE infants with serial MRS on DOL 5–6 immediately before (trough = 0 h), and after NVD (peak = 12–30 min) and delayed (post-peak = 2–6 h), with NAC alone (*n* = 5) or NVD infusion (*n* = 5). (**B**) [GSH] change with NAC 25 mg/kg/dose or 40 mg/kg/dose from trough to peak [GSH] or peak to post-peak (2–6 h) after NAC/NVD infusion (* *p* < 0.05). We included all patients who had the entire dose reliably delivered prior to second scan (efficacy analysis). (**C**,**D**) Trough to peak increase in plasma [NAC] plotted with the simultaneous increase in basal ganglia metabolites (**C**) [GSH] and (**D**) [tCR] for the 17 subjects with adequate MRS and [NAC] plasma data.

For the 10 infants with three scans, [GSH] showed a significant increase acutely and over time (*F* = 8.2, *p* = 0.012, partial eta sq = 0.67). Seven out of 10 infants maintained improved [GSH] in BG for four to six hours (Figure 3A). We observed a CNS dose response with a significantly greater increase in BG [GSH] with NAC 40 mg/kg (+32%) versus 25 mg/kg (+18%, *p* < 0.05, efficacy analysis, Figure 3B). Importantly, peak [GSH] in BG did not exceed the normal range in healthy term neonates with either NAC dose [52]. We previously reported CNS [GSH] in the BG increased significantly 12–30 min after NAC/NVD (*p <* 0.0001, *n* = 23, all doses, intent-to-treat, paired *t*-test), which was not significantly different between NAC alone (*n* = 9) vs. NVD (*n* = 14) [15]. Coefficients of variance were ≤8% for [GSH] with LCModel processing of STEAM sequences at TE20ms on Siemens 3T MRI. 

#### 3.4.2. Total Creatine and Choline Change Acutely with NVD Dosing 

NVD (all doses) significantly increased tCr, tCho, and mIns within 12–30 min of infusion by paired *t*-test (*n* = 23–24 depending on metabolite, intent to treat, Table 3). Mean NAA increased from pre- to immediately post-NVD (*n* = 24, intent to treat, *p* = 0.051, paired *t*-test), which was significant when infants with infusion problems were excluded (*n* = 18, *p* = 0.042). There was a trend to a dose response for [tCr] and [tCho] in the BG, with greater increase with NAC 40 mg/kg versus 25 mg/kg (both *p = 0.07*). 

For the 10 subjects with three scans, other metabolite concentrations were significantly different over the three scans: tCr (*F* = 10.9, *p =* 0.005, partial eta sq = 0.73); tCho (*F* = 20.9, *p* = 0.001, partial eta sq = 0.83); mIns (*F* = 9.1, *p* = 0.009, partial eta sq = 0.7). There were no significant differences in [NAA] or [GLX] within these 10 subjects by repeated measures ANOVA. 

#### 3.4.3. Sex Differences in CNS Metabolites

[GSH] in BG before NVD infusion showed a strong tendency to be lower in HIE males (1.59 ± 0.19 mM, *n* = 17) than in females (1.74 ± 0.18 mM, *n* = 6, *p* = 0.06), but [GSH] increase with NVD was not different by sex. There were no sex differences in mean metabolite concentrations for other metabolites, but there were too few females for meaningful repeated measures comparisons. 

### 3.5. Plasma NAC and Lipid Peroxides Correlate with CNS Markers of Oxidative Stress and Energetics

**Figure 4:**
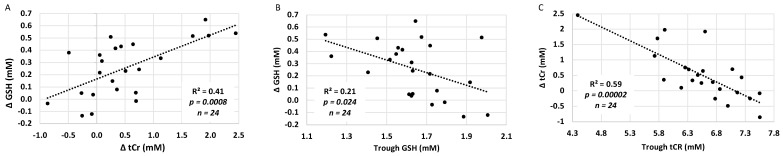
Comparison of GSH and tCr in BG (intent to treat analysis). (**A**) The increase in [GSH] is plotted against the change in [tCR] trough to peak, across all infants with NAC/NVD infusion, including those with only partial infusions. (**B**,**C**) The increase in (**B**) [GSH] and (**C**) [tCr] are strongly dependent upon the trough concentrations in the BG. There was a significantly greater increase in [GSH] and [tCR] in those with lower CNS GSH and tCr on DOL 5 before dosing (trough).

NAC plasma concentrations strongly correlated with the simultaneous increase in CNS [GSH] and [tCr] from trough to peak, before and after the NVD infusion (Figure 3C,D). The absolute change in [ΔGSH] also positively correlated with the absolute change in [ΔtCr] from trough to peak (Figure 4A), demonstrating that CNS energetics improved in conjunction with antioxidant concentrations. Moreover, the lower the baseline [GSH] and [tCr] before dosing (trough), the greater the increase with NAC/NVD (Figure 4B,C). Conversely, the higher the trough [GSH] and [tCr] in BG before NAC/NVD, the less the absolute increase in [GSH] and [tCr].

**Figure 5:**
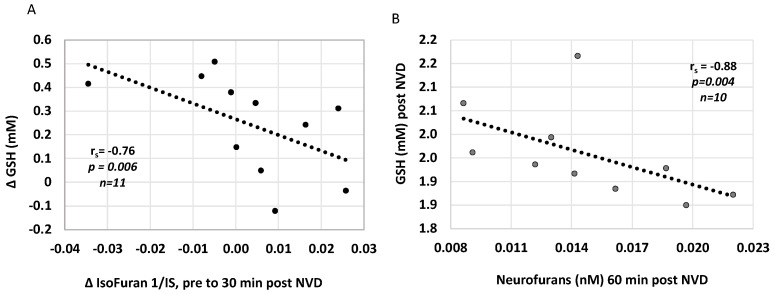
Plasma oxidative stress markers Isofurans and Neurofurans correlate with CNS [GSH] after NVD. (**A**) The increase in [GSH] is strongly correlated with a decrease in Isofurans from pre to post NVD dosing. (**B**) [GSH] after NVD infusion is strongly negatively correlated with Neurofurans at 60 min post infusion. (Spearman’s rank correlation).

We also investigated whether plasma concentrations of lipid peroxidation markers, isoprostanoids, were associated with CNS oxidative stress and acutely changed with NAC/NVD infusion in paired plasma samples around DOL 5 MRS. The decrease in Isofuran concentrations from before to immediately after NAC/NVD infusion correlated with simultaneous increase in CNS [GSH], from trough to 30 min peak (*r* = −0.76, *p* = 0.006, *n* = 11, Figure 5A). Several plasma oxidative stress markers were inversely correlated with CNS [GSH] post NVD. Higher CNS [GSH] correlated with lower plasma Neurofurans at one hour post NVD (*r*_s_= −0.88, *p* = 0.0038, *n* = 10, Figure 5B); Other correlations were limited due to small numbers of samples but include 5IPF2a-VI concentrations at 30 min after NVD dosing (*r*= −0.78, *p* = 0.0022, *n* = 8). Neither 25- or 1,25(OH)_2_ plasma concentrations correlated with plasma iso- or neurofurans.

### 3.6. Death and Neurodevelopmental Outcomes

The mean days to discharge for 26 HIE infants (excluding prolonged ECMO or genetic syndromes) was 16 ± 12 d. There were no deaths during initial hospitalization. One infant suffered a roll-over accidental death at three months during co-bedding. The two infants with a history of ECMO and one infant with a genetic syndrome were the only participants to require gastrostomy tubes for feeding. The ECMO and genetic syndrome patients had abnormal development with spastic diplegic cerebral palsy (1), global developmental delay (4), and autism (1). One infant was lost to follow-up. 

The remaining 24 infants with HIE treated with NVD and hypothermia show normal gross motor, fine motor, and cognitive development at 24–48 months of age, without evidence of cerebral palsy or autism. These 24 infants include 10 infants either exposed to chorioamnionitis or diagnosed with clinical sepsis and pneumonia. Twenty-two had formal developmental assessments at developmental follow-up clinics: the mean Peabody gross motor developmental quotient was 102 ± 9.5, the Cognitive adaptive test (CAT) was 95 ± 9.3, the Clinical linguistics and auditory milestone scale (CLAMS) was 98.9 ± 11.1, and the mean MCHAT was 0.4 ± 0.5, at 33 ± 14 months of age. In the severe HIE group (*n* = 13), the Peabody gross motor developmental quotient was 103 ± 8, CAT 101 ± 10, CLAMs 95 ± 11, MCHAT 0.14 ± 0.4 at 29 ± 12 months of age. Three male infants were receiving therapy for speech delay, and one made significant improvement after myringotomy tube placement. Parents of two infants who moved out of the area reported normal development and no special services. 

## 4. Discussion

In neonates with moderate and severe HIE, oxidative stress continues for days after completion of therapeutic hypothermia, as reflected in low concentrations of reduced glutathione in the basal ganglia before NVD dosing on DOL 5–6. NAC crossed the blood–brain barrier quickly and significantly increased [GSH] in the BG of HIE neonates on DOL 5–6, in a dose responsive manner, with a measurable effect for at least 6 h, while the serum NAC half-life during the same normothermic period was 4.5 h. Both NAC doses resulted in GSH increases, but the 40 mg/kg dose resulted in a 32% increase within 30 min of infusion. The 40 mg/kg dose of NAC appeared safe both systemically and in CNS, as we did not observe evidence of reductive stress [63], and GSH never exceeded reported normal values in term infants.

At the same time, NAC/NVD increased total creatine in the BG, which is strongly correlated with the increase in [GSH]. The simultaneous rapid improvement in tCr indicates that along with restoring CNS GSH reserves, NAC and or NVD improve mitochondrial and cellular creatine kinase and synthetic function [64] and increase important energy substrates in a vulnerable brain region. Further, the increases in GSH and tCr were greater in patients with lower total creatine and greater oxidative stress, and not as great in infants with higher GSH or tCr concentrations. Taken together, these data strongly suggest that NAC 25–40 mg/kg/dose and calcitriol result in rapid synthesis of GSH and increases in CNS energetics that are titrated based on level of CNS demand from oxidative stress in the BG. 

This finding is immensely important for the safe clinical translation of these therapeutics. Under normal conditions, the production of intracellular GSH is tightly regulated by availability of the rate-limiting precursor cysteine and direct feedback inhibition of the major synthetic enzyme, glutamate-cysteine ligase [65]. In hypoxic-ischemic and reperfusion conditions, the feedback inhibition via phosphorylation may be impaired, leading to excess GSH and reductive stress even with low doses of NAC and vitamin D. Our MRS data on DOL 5 show that feedback inhibition is intact and there is tight regulation of GSH synthesis to a normal intracellular concentration. Therefore, infants with the greatest oxidative stress can benefit from NVD, without exposing less affected infants to harmful side effects.

Plasma isofurans, which are stable and peak more slowly than isoprostanes [66], were also reduced after NAC/NVD infusion on day five, which strongly correlated with the simultaneous increase in CNS [GSH]. The combined data on CNS and plasma oxidative stress markers indicate that infants are still not metabolically stable during the subacute phase of HIE despite hypothermia treatment, and that NAC rapidly corrects both systemic and CNS oxidative stress and metabolomics. Even infants with severe HIE by neurologic exam, cord pH ≤ 6.8, and MRI lesions seemed to benefit, as motor and cognitive outcomes for this group were within the normal range. The fact that significant structural MRI lesions were found on DOL 5 in one perinatal arterial stroke patient and yet functional outcomes at 24 months of age were favorable without hemiparesis, suggests that this metabolic recovery is very important to functional outcome. 

NAC and vitamin D pharmacokinetics differ by hypothermic and normothermic phases of recovery. Similar to findings of other PK trials in neonatal therapeutic hypothermia [67,68], we found longer t_1/2_ and decreased CL of NAC during hypothermia when compared to normothermia. Based on this data, NAC dose and dosing interval need to be adjusted to increase NAC trough steady state concentrations during both HT and NT. NAC 30 mg/kg every eight hours and 40 mg/kg every eight hours during HT and NT, respectively, will maintain troughs above 61 mm/L (10 mcg/mL) and peaks below 613.5 mm/L (100 mcg/mL). The pairing of blood samples with MRS scans provides a unique dataset that directly links intravenous administration of NVD to target effects in a critical CNS region and identify NAC doses and plasma levels that alleviate a key mechanism of oxidative stress and compromised CNS energetics after HIE birth. These data provide important dosing information for future clinical trial design.

We were not as successful in identifying a direct CNS target of 1,25(OH)_2_D effect, as vitamin D’s effect did not manifest as acute changes by serial MRS. However, we again demonstrate that the majority of HIE neonates are vitamin D insufficient and the standard 400IU supplemental dose of 25(OH)D is inadequate to correct this insufficiency, as we described in our previous RCT trial of hypothermia [57]. Our studies and other studies of vitamin D deficiency in HIE highlight a significant unmet need for these critically ill infants with neuro- and systemic inflammation [69,70]. Severe vitamin D deficiency is well described to impart a significant risk for worse outcomes after stroke in adult humans and animal models [71,72,73]. Additionally, in neonatal HIE, lower 25(OH)D concentrations in the first 48 h inversely correlated with MRI evidence of brain injury [70]. Conversely, stroke results in lower vitamin D levels through inflammation-triggered degradation of vitamin D [16,74]. Vitamin D has been shown to be neuroprotective when given primarily before and, in one study, after ischemia reperfusion injury [38,47,75,76]. 1,25(OH)_2_D may act by regulating NMDA receptors and protecting against glutamate excitotoxicity [42,76], reducing inflammatory cytokines, modulating regulatory T cells [16,77], or chronically upregulating GSH synthetic enzymes and increasing GSH production [42,43,44]. While the evidence in general suggests that vitamin D deficiency appears to be detrimental for adult brain after stroke, vitamin D deficiency after global stroke in neonates may be particularly impactful, given vitamin D’s important effects on plasticity, cognition and memory during brain development [16,38,39,40,78,79]. Based on our finding and those of others we suggest that all HIE patients have their vitamin status determined on admission and treated appropriately to achieve a serum 25(OH)D > 50 nmol/L. 

Our study revealed mildly elevated ionized calcium even with q24h calcitriol dosing, which was managed by decreasing intravenous calcium or holding calcitriol doses. While we saw no clinical adverse effects from this biochemical finding, we were only able to complete an average of six out of ten days of calcitriol dosing per our safety protocol. As 25(OH)D serum concentrations had not reached sufficiency by the end of the study, increased 25(OH)D supplementation should be considered upon reaching seven days, full oral feeds, or discharge from the hospital. 

We used much lower doses of NAC than employed in acetaminophen toxicity protocols or in maternal inflammation animal models of neonatal brain injury (200–300 mg/kg) [8,9,25,80,81]. Results from our trial confirm our NAC dose selection based on our preclinical studies [14,16,37,82], and demonstrate desired effects on CNS and systemic oxidative stress with NAC 25–40 mg/kg in HIE neonates without significant side effects. NAC appears to be safe in critically ill HIE neonates, just as we demonstrated in neonates of all gestational ages exposed to maternal intrauterine inflammation in our trial of NAC in maternal chorioamnionitis [83,84]. In addition to our clinical study, two other preclinical studies have demonstrated inhibition of peroxynitrite and reduction of oxidative stress with NAC 20–40 mg/kg [25,80].

We observed no significant motor, cognitive or behavioral deficits in the 24 HIE infants in pediatric follow-up, particularly impressive considering six infants had cord pH 6.5–6.8, one with no detectable heart rate until 25 min of life. Based on historical and more recent studies of hypothermia outcomes, we would expect between 8–12 infants in our cohort to have significant disability at two to four years of age [85,86]. This clinical trial extends our preclinical studies of the neuroprotective effects of NVD and demonstrates a likely neuroprotective effect in HIE neonates with severe HIE (53%) and chorioamnionitis (42%) [14,16]. We have previously shown that oxidative stress is still increasing after two hours of hypothermia in a lipopolysaccharide-HI animal model, indicating that hypothermia acts too slowly to affect this severe injury [14]. In contrast, NAC and 1,25(OH)2D rapidly corrected glutathione and suppressed glutamate in these hypothermic LPS-HI rat pups. Taken together, these preclinical and clinical studies indicate that NAC and vitamin D can mitigate overwhelming oxidative stress, treating gaps in hypothermic neuroprotection that occur both early during severe HI injury and later in the convalescent phase.

We describe a strong trend to lower [GSH] in males than in females on DOL 5, but we note that sexes were unbalanced in our cohort, with twice as many males as females, reducing our ability to accurately evaluate sex differences. However, other investigators have noted sex differences with greater oxidative stress and neural damage in males or male neurons after HI than in females, as we observed in our preclinical studies, in which low dose NAC alone did not neuroprotect males [30,87,88,89].

This study adds to other work demonstrating that MRS is a quantitative translational method for measuring neural injury (NAA, tCr) and antioxidant status (GSH) in HIE neonates and adults with stroke and demonstrates the unique ability to assess direct therapeutic response in a highly susceptible area of the CNS in clinical trials [15,86,90]. MRS with NAA, lactate, choline and creatine metabolite ratios are less reliable as pharmacodynamic markers, as both numerator and denominator change rapidly after stroke with their own time courses [19,90,91]. We demonstrate that serial [GSH] by MRS generates a CNS-specific, quantitative, PD time-course of treatment effect, informs effective dosing of antioxidants for a target CNS tissue/tract, and increases the likelihood of attaining the primary endpoint in subsequent clinical trials. As MRS measurements of NAA provide the best prognostic biomarker for prediction of disability early after HI injury [86,92,93], a simplified MRS protocol with a single voxel in the BG at low TE captures both GSH and NAA and would be feasible for phase II clinical trial endpoints. 

This work has several limitations, chiefly the smaller sample size in some groups. We could not draw normothermia blood samples for 1,25(OH)_2_D PK when calcitriol doses were held or were timed differently (q24h) than NAC doses, and did not coincide with the MRI scans, which had to be conducted at night. This resulted in fewer samples for 1,25(OH)_2_D PK. We also had 1,25(OH)_2_D serum concentrations that fluctuated and prevented PK estimate calculations and comparisons between dosing groups. We did not have MRS data immediately after HIE birth, and thus cannot quantify CNS oxidative stress in the earliest phase of injury. Our MRS scans were not optimized for lactate, but rather for GSH, and showed large coefficients of variation on LC Model spectral fitting of lactate, indicating poor confidence in isolation of lactate peaks from overlapping macromolecular and lipid peaks. We did not have funding for Bayley developmental testing and relied on other validated tests that are strongly correlated with the Bayley and are routinely performed in our high-risk clinic, as well as parent responses for two infants who had moved from the area. 

## 5. Conclusions

In summary, in this prospective trial of NAC and 1,25(OH)_2_D in hypothermic HIE neonates, we provide rigorous data demonstrating that CNS oxidative stress and compromised CNS energetics persist during the first week after hypoxia ischemic birth, and that NAC 25–40 mg/kg/dose and calcitriol result in rapid synthesis of GSH and increases in CNS energetics that are titrated based on the level of CNS demand from oxidative stress in the BG. With the favorable neurodevelopmental outcomes in this human trial and our preclinical animal studies, these data provide important dosing information for future clinical trial design and a strong basis for a randomized trial of NAC and 1,25(OH)_2_D in HIE neonates.

## Figures and Tables

**Table 1:** Demographics of HIE infants. Mean (SD) or number of subjects with specific conditions.

	*N* = 30
Gestational age (weeks), mean (SD)	38.2 ± 1.5
Birth weight (grams), mean (SD)	3248 ± 545
Males/Females	20/10
pH of cord or neonatal blood, median (IQR)	6.91 (6.82, 6.99)
Cord pH 6.5–6.8, number of subjects	6
Base deficit of cord or neonatal blood, median (IQR)	−18.0 (−20, −13.6)
Base deficit ≥ −20, number of subjects	10
Serum lactate (mM) < 6 h after birth, mean (SD)	8.9 ± 4.6
Apgar score at 1 min, mean (SD)	1.4 ± 1.3
Apgar score at 5 min, mean (SD)	3.2 ± 2.0
Apgar score at 10 min, mean (SD)	5.4 ± 2.1
Chest compressions, number of subjects	10
HIE stage 2/3, number of subjects	14/16
Placental histology: Chorioamnionitis	8/19 (42%)
Funisitis	6/19 (32%)
Clinical Sepsis/Pneumonia	8
Complete abruption or feto-maternal hemorrhage	5
Seizures	7
PPHN	6
ECMO	2
Congenital syndrome	2
MRI abnormalities, total number of patients	13
Minor T1 & T2 signal abnormalities	10
Basal ganglia	1
Periventricular white matter	7
Cortical/subinsular	1/1
Hemorrhages	
Periventricular/Cortical/Occipital/Cerebellum	1/1/1/1
Perinatal arterial stroke	1
Gr 1–2 IVH	4

**Table 2:** NAC and 1,25(OH)2D PK during HT and NT.

NAC PK	**GA** **wks**	**Birth** **Wt gm**	**t_1/2_ (hrs)** **HT vs. NT**		**Vd (L/kg)** **HT vs. NT**		**CL (mL/hr/kg)** **HT vs. NT**		**Cmin_ss_ µMol** **HT vs. NT**		**Cmax_ss_ µMol** **HT vs. NT**		**AUC 0-t** **HT vs. NT**	
Mean NAC 25 mg/kg (SD)	38.1 (1.6)	3210 (456)	5.8 (1.9)	4.2 (1.1)	0.63 (0.29)	0.73 (0.31)	68 (23)	113 (35)	70.8 (54.8)	25.6 (23.6)	365.0 (93.2)	251.1 (84.0)	300.4 (71.8)	200.1 (69.6)
*p* value HT vs. NT NAC 25 mg/kg			0.0050 *		0.1415 *		0.0006 *		0.0017 *		0.0030 *		0.0018 *	
Mean NAC 40 mg/kg (SD)	38.8 (1.3)	3110 (1137)	4.9 (1.3)	4.0 (1.3)	0.49 (0.13)	0.70 (0.1)	70 (16)	133 (41)	105.8 (49.1)	47.9 (36.8)	585.9 (117.8)	365.6 (71.2)	467 (96.6)	265.7 (71.7)
*p* value HT vs. NT NAC 40 mg/kg			0.15 *		0.0026 *		0.0018 *		0.0208 *		0.0019 *		0.0021 *	
*p* value NAC 25 vs. 40 mg/kg/dose											0.12	0.07	**0.0018**	**0.0018**
1,25(OH)_2_ D PK	**GA** **wks**	**Birth** **Wt gm**	**t_1/2_ (hrs)** **HT vs. NT**		**Vd (L/kg)** **HT vs. NT**		**CL (mL/hr/kg)** **HT vs. NT**		**Cmin_ss_ pMol** **HT vs. NT**		**Cmax_ss_ pMol** **HT vs. NT**		**AUC 0-t** **HT vs. NT**	
Mean 1,25(OH)_2_ D 0.05 mcg/kg	37.9 (1.4)	3197 (466)	26.2 (16.0)	18.3 (7.0)	0.65 (0.25)	0.43 (0.45)	24.4 (15.5)	14.1 (11.8)	458 (242)	737 (742)	648 (238)	1330 (1445)		

* Indicate comparisons of HT and NT PK parameters within dosing group.

**Table 3:** MRS metabolite concentrations for all subjects, Mean (SD).

	**Acute MRS (DOL 5)**			**Convalescent MRS**
CNS metabolite (mM)	**Pre-NVD** **Trough (*n* = 24)**	**Post-NVD** **Peak (*n* = 24)**	**Post-NVD** **Post-Peak (*n* = 10)**	**DOL 11–40** **(*n* = 18)**
GSH	1.61 ± 0.28 *	1.93 ± 0.31 *	1.77 ± 0.32	2.05 ± 0.37
Total Creatine	6.49 ± 0.7 *	7.06 ± 0.53 *^,†^	6.98 ± 0.81 ^†^	6.93 ± 0.55
Total Choline	2.43 ± 0.3 ^‡^	2.67 ± 0.3 *^,‡^	2.46 ± 0.30 *	2.63 ± 0.33
NAA	4.62 ± 0.5	4.84 ± 0.46	4.55 ± 0.71	5.11 ± 0.81
Glutamate + glutamine	6.59 ± 0.94	6.37 ± 0.57	7.04 ± 0.74	7.35 ± 1.08
MyoInositol	7.12 ± 0.95 ^†^	7.65 ± 0.91 ^†^	7.13 ± 0.70	7.83 ± 0.97

Comparing MRS metabolites over 3 scans during the acute HIE phase, before and after NVD infusion on DOL5, intent to treat analysis: overall ANOVAs are significant for GSH (*p* = 0.012), tCr (*p* = 0.005), tCho (*p* = 0.001); mIns (*p* = 0.009). Specific groups that were significantly different by pairwise comparisons are noted by * *p* < 0.05, ^†^ *p* < 0.005, ^‡^ *p*< 0.0005.

## Data Availability

Individual NAC and vitamin D PK data are available in Supplemental data. Raw MRS data were generated at MUSC. Derived MRS data supporting the findings of this study are available with reasonable request.

## Supplementary Materials

The following are available online at https://www.mdpi.com/article/10.3390/antiox10091344/s1, Table S1. NAC PK (25 & 40 mg/kg/dose) for individual participants; Table S2. 1,25(OH)_2_D (0.05 mcg/kg/dose) PK for individual participants.

## Abbreviations

AUC = area under the curve; BG = basal ganglia; CL = clearance; Cmax_ss_ = concentration maximum at steady state; Cmin_ss_ = concentration minimum at steady state; DOL = day of life; ECMO = extracorporeal membrane oxygenation; FAS-FAS cell surface death receptor; GC-MS = gas chromatography-mass spectrometry; GSH = reduced glutathione; GLX = glutamate + glutamine; HI = hypoxia ischemia; HIE = hypoxic ischemic encephalopathy; HPLC = high pressure liquid chromatography; HT = hypothermia; IRB = Institutional Review Board; IVH = intraventricular hemorrhage; mIns = myoinositol; MRS = magnetic resonance spectroscopy; NAA = N-acetylaspartate + N-acetylaspartylglutamate; NAC = N-acetylcysteine; NMDA = N-methyl-D-Aspartate; NT = normothermia; NVD = NAC + vitamin D (calcitriol); PD = pharmacodynamic; PK = pharmacokinetic; PPHN = persistent pulmonary hypertension; PRESS = single point resolved spectroscopy; RIA = radioimmunoassay; ROS = reactive oxygen species; STEAM = stimulation echo acquisition mode; t_1/2_ = half-life; TE = echo time; tCho = glycerophosphocholine + phosphocholine; tCr = creatine + phosphocreatine; Vd = volume of distribution.
