# Supplementary material for: NAC and Vitamin D Improve CNS and Plasma Oxidative Stress in Neonatal HIE and Are Associated with Favorable Long-Term Outcomes
Source: Antioxidants (Basel). 2021 Aug 25;10(9):1344. doi: 10.3390/antiox10091344 (PMC8466838; doi:10.3390/antiox10091344)
Supplement: Supplementary file 1 [file antioxidants-10-01344-s001.zip › antioxidants-1329322-supplementary.pdf]

Table S1. NAC PK (25 & 40mg/kg/dose) for individual participants

ID-NAC dose	GA (wks)	Birth Weight (gm)	$t_{1/2}$ (hrs) HT vs NT		Vd (L/kg) HT vs NT		CL (mL/hr/kg) HT vs NT		NAC $Cmin_{ss}$ (mcg/mL) HT vs NT		NAC $Cmax_{ss}$ (mcg/mL) HT vs NT		NAC CSF (mcg/mL)	AUC HT vs NT	
**NAC 25mg/kg**															
NVD-1-25	37.8	3125	3.6	1.9	1.26	1.46	78	173	5.7	0.5	58.0	41.6	0.74	296	143
NVD-2-25	41.0	3430	4.2	2.3	0.41	0.48	68	142	7.5	1.0	55.3	34.4		277	151
NVD-3-25	37.3	3615	8.3	3.8	0.65	0.59	54	107	21.6	4.6	59.1	41.7		266	201
NVD-4-25-ECMO	36.0	3180	6.1	5.8	0.78	0.42	89	51	10.5	17.3	41.5	72.7		188	365
NVD-5-25	37.0	2130	6.6	4.5	0.57	0.73	59	112	16.6	5.7	58.4	36.5		285	185
NVD-6-25-ECMO	37.9	2645	4.5	5.8	0.76	1.13	117	133	3.0	1.9	77.5	22.5		437	144
NVD-7-25	36.0	3560	5.3	3.3	0.41	0.48	54	101	16.3	4.4	79.3	54.9		389	235
NVD-8-25	40.4	3380	4.4	3.7	0.47	0.64	73	121	8.8	4.1	57.4	39.8		284	182
NVD-9-25	37.0	2370	5.7	5.2	0.48	0.52	58	69	4.2	3.0	52.8	48.8		312	273
NVD-10-25	41.1	3380	8.1	4.0	0.49	0.63	42	111	28.0	5.1	78.3	41.7		359	183
NVD-12-25	36.9	3440	6.0	5.0	1.36	0.82	130	114	2.0	1.6	22.5	29.5		138	174
NVD-13-25	39.7	3824	4.0	5.7	0.40	0.77	69	94	1.5	2.6	56.5	33.1		310	196
NVD-14-25	40.0	3581	3.9	3.9	0.47	0.56	83	99	1.2	1.0	47.5	39.2		259	223
NVD-15-25	39.1	3430	5.8	3.2	0.56	0.32	67	68	3.7	0.7	45.4	64.3		263	334
NVD-16-25	36.7	3080	5.8	3.7	0.49	0.49	58	92	14.9	5.5	63.0	52.0		316	221
NVD-24-25	37.6	3050	5.8	4.7	0.40	1.30	48	190	17.4	3.5	72.7	20.4	1.32	368	100
NVD-25-25	38.0	2910	4.9	5.1	0.33	0.73	47	100	15.1	7.4	82.8	37.8		387	188
NVD-28-25	37.9	3645	11.2	4.8	0.76	1.03	47	149	29.8	4.7	62.5	26.6	9.76	274	104
**Mean (SD) NAC 25mg/kg**	**38.1<br>(1.6)**	**3210<br>(456)**	**5.8<br>(1.9)**	**4.2<br>(1.1)**	**0.63<br>(0.29)**	**0.73<br>(0.31)**	**68<br>(23)**	**113<br>(35)**	**11.5<br>(8.9)**	**4.1<br>(3.8)**	**59.5<br>(15.2)**	**41.0<br>(13.7)**		**389<br>(102)**	**238<br>(86)**
p value*<br>HT vs NT NAC25mg/kg			**0.0050***		0.1415		**0.0006***		**0.0017***		**0.0030***				
**NAC 40mg/kg**															
NVD-17-40	39	3325	7.1	3.8	0.78	0.77	77	144	21.8	5.6	70.5	51.4		341	241
NVD-18-40	38.3	3380	3.8	3.5	0.55	0.68	101	132	7.8	5.2	69	54.6		344	268
NVD-19-40	40.2	3238	3.6	2.3	0.40	0.71	78	213	9.1	1.3	93	47.5	0.52	453	180
NVD-20-40	38.9	3495	4.2	4.3	0.40	0.67	66	107	14.4	9.8	105.6	67.2		497	295
NVD-21-40	40.4	3470	3.9	6.2	0.43	0.63	77	71	11.4	21.4	96.3	82.4	1.60	418	409
NVD-22-40	38.3	3745	6.3	5.5	0.42	0.93	46	117	33.6	10.4	127	47.4		619	275
NVD-27-40	39.0	4005	4.8	4.5	0.39	0.68	56	105	19.6	9.5	112.5	60.1		571	319
NVD-29-40	36.0	2445	4.5	3.1	0.41	0.59	63	135	16.2	4.5	103.3	69.2		534	221
NVD-30-40	39.0	3930	6.2	2.8	0.61	0.69	69	173	21.4	2.6	82.5	56.5		426	183
**Mean (SD) NAC 40mg/kg**	**38.8<br>(1.3)**	**3110<br>(1137)**	**4.9<br>(1.3)**	**4.0<br>(1.3)**	**0.49<br>(0.13)**	**0.70<br>(0.1)**	**70<br>(16)**	**133<br>(41)**	**17.3<br>(8.0)**	**7.8<br>(6.0)**	**95.5<br>(19.2)**	**59.6<br>(11.6)**		**573<br>(160)**	**325<br>(111)**
p value* HT vs NT<br>NAC40mg/kg			0.15		**0.0026***		**0.0018***		**0.0208***		**0.001***				
p value NAC 25 vs 40mg/kg									0.12	0.07	**0.0018**	**0.0018**			

Table S2. 1,25(OH)$_2$D (0.05mcg/kg/dose) PK for individual participants

ID	GA (wks)	Birth Weight (gm)	$t_{1/2}$ (hrs) HT vs NT		Vd (L/kg) HT vs NT		CL (mL/hr/kg) HT vs NT		$Cmin_{ss}$ (μmol/L) HT vs NT		$Cmax_{ss}$ (μmol/L) HT vs NT	
NVD-1	37.8	3125	29.8		0.88		20.5		415		550	
NVD-2	41.0	3430	57.3		0.84		10.2		943		1,090	
NVD-3	37.3	3615	15.6		0.35		19.2		367		712	
NVD-4-ECMO	36.0	3180	19.0		0.84		31.0		264		408	
NVD-8	40.4	3380	20.6	13.3	0.64	0.11	22.0	6.0	377	1,260	564	2,352
NVD-13	39.7	3824	20.5	23.3	0.36	0.75	12.2	22.4	379	211	571	307
**Mean**	**37.9**	**3197**	**26.2**	**18.3**	**0.65**	**0.43**	**24.4**	**14.1**	**458**	**737**	**648**	**1,330**
**(SD)**	**(1.4)**	**(466)**	**(16.0)**	**(7.0)**	**(0.25)**	**(0.45)**	**(15.5)**	**(11.8)**	**(242)**	**(742)**	**(238)**	**(1,445)**
